# Quercetin and Rosmarinic Acid Functionalized Hybrid Electrospun Nanofibers with Strong Antioxidant and Anticancer Activities

**DOI:** 10.3390/biomimetics11070453

**Published:** 2026-07-01

**Authors:** Nikoleta Stoyanova, Nasko Nachev, Ani Georgieva, Reneta Toshkova, Mariya Spasova

**Affiliations:** 1 Laboratory of Bioactive Polymers, Institute of Polymers, Bulgarian Academy of Sciences, Akad. G. Bonchev St., Bl. 103A, 1113 Sofia, Bulgaria; nstoyanova@polymer.bas.bg (N.S.); nachev_n@polymer.bas.bg (N.N.); 2 Centre of Competence “Sustainable Utilization of Bio-Resources and Waste of Medicinal and Aromatic Plants for Innovative Bioactive Products” (BIORESOURCES BG), 1000 Sofia, Bulgaria; 3 Institute of Experimental Morphology, Pathology and Anthropology with Museum, Bulgarian Academy of Sciences, Acad. G. Bonchev St, Bl. 25, 1113 Sofia, Bulgaria; georgieva_any@abv.bg (A.G.); reneta.toshkova@gmail.com (R.T.)

**Keywords:** poly(L-lactic acid), PEG, polyphenols, mechanical properties, antioxidant activity, HeLa cervical cancer cells, BALB/3T3 fibroblasts

## Abstract

In this study, novel electrospun polymer mats based on biocompatible poly(lactic acid) (PLA) and hydrophilic poly(ethylene glycol) (PEG) were successfully fabricated for the co-delivery of two natural polyphenols, quercetin (QUE) and rosmarinic acid (RA). Scanning electron microscopy (SEM) revealed the formation of defect-free, continuous nanofibers with high interconnected porosity. By mimicking the structural features of the native extracellular matrix, these nanofibrous platforms facilitate pronounced combined antioxidant and anticancer action. X-ray diffraction (XRD) analysis confirmed that the rapid solvent evaporation during electrospinning induced a physical state transformation, converting both QUE and RA from their native crystalline structures into an amorphous dispersion within the polymer fibrous materials, thereby optimizing their potential bioavailability. The obtained hybrid fibrous materials possessed good mechanical properties. Moreover, the 2,2-diphenyl-1-picrylhydrazyl (DPPH) radical scavenging assay demonstrated that the incorporation of PEG enhanced matrix hydrophilicity, allowing the four-component PLA/PEG/QUE/RA mats to achieve the highest antioxidant efficiency (98.1%), suggesting an enhanced, complementary radical-neutralization pathway. Furthermore, in vitro biological assessments against human cervical carcinoma cell line (HeLa) and normal murine embryo fibroblasts BALB/3T3 demonstrated prominent anticancer activity, while noncancerous cells were significantly less affected. The dual-loaded PLA/PEG/QUE/RA fibrous mats induced significant cell shrinkage, chromatin condensation, and apoptotic cell death in HeLa cells, while normal BALB/3T3 fibroblasts retained cell membrane integrity and displayed higher resistance. Modeled after the native extracellular matrix, these bioinspired materials demonstrate significant antioxidant and anticancer activity, highlighting their potential for applications in localized cancer therapy, wound management, and tissue engineering.

## 1. Introduction

Due to their promising medicinal properties, phytochemicals—naturally occurring bioactive substances in plants—have become a major focus of scientific investigation [1]. Among these, polyphenols, characterized by multiple phenolic hydroxyl groups, represent a major class with notable antioxidant [2], anti-inflammatory [3], and anticancer [4] activities. This is owing to their remarkable capacity to regulate oxidative stress [5], inflammatory processes [6], and intracellular signaling pathways [7]. Within the vast array of plant-derived bioactive molecules, quercetin (QUE) and rosmarinic acid (RA) hold promise as particularly strong candidates by virtue of their well-established pleiotropic biological activities [8,9]. QUE, a dietary flavonoid from many fruits and vegetables, possesses antioxidant [10], anti-inflammatory [11], and anticancer [12] activity by neutralizing free radicals [13], inhibiting enzymes [14], and modulating apoptotic pathways [15]. RA, a naturally occurring hydroxycinnamic acid ester predominantly found in medicinal herbs such as rosemary and lemon balm, demonstrates exceptional radical-scavenging capacity [16] and has been extensively studied for its neuroprotective [17], hepatoprotective [18], and antiproliferative [19] effects. Combining these polyphenols may have synergistic benefits, particularly for conditions involving chronic inflammation and oxidative stress, potentially improving therapeutic outcomes compared to single-agent administration. The development of formulations containing QUE and RA is complicated by the fact that both polyphenols exhibit limited dissolution in water-based systems. This physicochemical characteristic adversely affects their absorption and reduces the amount of bioactive compound available at the target site [20,21]. They are also unstable when exposed to light, heat, and pH changes, complicating their processing, storage, and in vivo application [22,23]. These challenges necessitate delivery platforms that can stabilize both compounds while providing controlled, targeted release. A powerful strategy to overcome these limitations lies in biomimetics—the design of synthetic materials that emulate time-tested structures and functions found in nature. Electrospinning is a flexible, scalable nanofabrication technique that produces polymeric fibrous matrices with biomimetic structural features, making them ideally suited for biomedical applications [24]. In the context of tissue engineering and localized drug delivery, the native extracellular matrix (ECM) serves as a particularly relevant biological blueprint. The ECM is a dynamic three-dimensional network of nanoscale fibrous proteins and glycoproteins that not only provides structural support but also regulates cell adhesion, proliferation, migration, and differentiation through its hierarchical architecture, mechanical properties, and biochemical cues. Consequently, reproducing key ECM characteristics, including fibrous morphology, interconnected porosity, and tissue-compatible mechanical behavior, has become a central objective in the development of advanced biomedical materials.

The resulting nanofibrous scaffolds possess exceptionally high surface-area-to-volume ratios, tunable porosity, and morphological similarities to the native extracellular matrix—characteristics that facilitate high drug loading efficiency [25], controlled release kinetics [26], and enhanced cellular interactions [27]. Moreover, electrospun fibers can be engineered to accommodate both hydrophobic and hydrophilic therapeutic agents within a single carrier system, offering significant potential for combined therapeutic regimens for multifaceted conditions like cancer [28].

Electrospun nanofibrous scaffolds are among the most successful biomimetic platforms because they closely resemble the structural organization of the native ECM [26]. Their high surface-area-to-volume ratio, tunable pore architecture, and nanoscale fiber dimensions create a microenvironment that supports cell–material interactions while simultaneously enabling efficient incorporation and controlled release of therapeutic agents [24].

The selection of appropriate polymer systems is critical to achieving desired release profiles, mechanical properties, and biological efficacy. Poly(L-lactic acid) (PLA) is commonly employed as a structural polymer; its favorable biocompatibility, biodegradability, and mechanical stability allow it to support the sustained release of encapsulated drugs [29]. However, the inherent hydrophobicity of PLA limits water penetration, necessitating the incorporation of hydrophilic components to optimize release behavior [30,31]. In this context, polyethylene glycol (PEG) enhances matrix wettability and water uptake [30], thereby facilitating diffusion-driven drug release [32]. Building upon our group’s extensive experience in developing electrospun polymer-based delivery systems for natural polyphenols, the present study represents a systematic advancement toward the rational design of multifunctional platforms for synergistic combination therapy. Our previous investigations have demonstrated that PLA-based electrospun fibers effectively encapsulate and protect quercetin, resulting in enhanced anticancer activity against various cancer cell lines while maintaining favorable biocompatibility profiles [33]. We have also established that the incorporation of PEG into PLA matrices significantly improves drug release kinetics and biological activity through enhanced wettability and accelerated diffusion [34,35]. Furthermore, our recent work has demonstrated that PLA/PEG composite systems can simultaneously incorporate and release both QUE and RA, resulting in synergistic anticancer effects against SH-4 melanoma cells with selective toxicity compared to normal HaCaT keratinocytes [33]. From a bioinspired standpoint, PLA/PEG-based electrospun fibers loaded with natural polyphenols represent an attractive example of how biodegradable polymers can be combined with naturally derived bioactive compounds to create multifunctional materials capable of mimicking biological tissues while delivering therapeutic signals for applications in localized cancer therapy, wound healing, and tissue regeneration. While previous studies have addressed morphological characterization, release kinetics, and preliminary biological evaluation using SH-4 and HaCaT cells, they have left several critical aspects unexplored. Advanced characterization by XRD, mechanical testing, solution rheology, antioxidant assay, and cell viability assays using different cell lines is needed in order to prove that the prepared complex materials are promising candidates for biomedical applications.

The current investigation seeks to address these knowledge gaps through a comprehensive and systematic characterization of PLA/PEG electrospun fibers co-loaded with QUE and RA, with particular emphasis on structural analysis, mechanical properties, and expanded biological evaluation. One distinctive aspect of the present study is the application of X-ray diffraction (XRD) analysis to elucidate the physical state of the incorporated polyphenols within the polymer matrices—a parameter of paramount importance given that amorphous drug forms demonstrate significantly higher dissolution rates and bioavailability relative to their crystalline counterparts [36]. We hypothesize that the rapid solvent evaporation inherent to the electrospinning process, combined with the molecular dispersion facilitated by the polymer matrix, will result in the conversion of QUE and RA from their native crystalline states to amorphous forms, thereby maximizing their therapeutic potential. Additionally, we extend our biological evaluation to include HeLa cervical cancer cells and normal BALB/3T3 fibroblasts, complementing our previous work on melanoma models and providing broader insights into the anticancer selectivity of these materials. The inclusion of comprehensive antioxidant activity assessment and detailed mechanical property evaluation further distinguishes this study from our prior contributions, enabling a more complete understanding of structure–property–function relationships.

The rationale for selecting both QUE and RA for co-delivery is underpinned by their complementary mechanisms of action and synergistic therapeutic potential. QUE displays multiple anticancer mechanisms, such as modulation of the cell cycle [15], caspase activation [37], and inhibition of survival signaling cascades [15], while RA contributes potent anti-inflammatory and pro-apoptotic activities that may enhance the overall antitumor efficacy [9,17]. Their combined antioxidant properties further support applications in oxidative stress-related conditions, including wound healing and tissue regeneration. By incorporating both compounds into a single PLA/PEG fibrous carrier, we seek to generate a two-stage release behavior characterized by rapid early release of bioactive molecules from the PEG-rich domains followed by a more gradual and prolonged release controlled by the PLA phase, thereby addressing both immediate and long-term therapeutic demands [38].

Accordingly, the present study was designed to pursue the following objectives: (i) to fabricate and comprehensively characterize PLA/PEG electrospun fibers co-loaded with QUE and RA, with emphasis on XRD analysis to confirm the amorphous state of the incorporated polyphenols; (ii) to evaluate the antioxidant activity and mechanical performance of the fabricated fibrous materials; and (iii) to investigate the biological response of HeLa cervical carcinoma cells and normal BALB/3T3 fibroblasts following exposure to the developed materials, thereby establishing the selectivity and therapeutic potential of this novel delivery platform. By integrating these analyses, we aim to provide a holistic understanding of how polymer composition, drug loading, and processing conditions influence material properties and biological performance, ultimately advancing the development of biomimetic electrospun polyphenol-loaded systems for applications in localized cancer therapy, wound management, and tissue engineering.

## 2. Materials and Methods

### 2.1. Materials

PLA (poly(lactic acid), grade Ingeo™ Biopolymer 4032D) with a molecular weight of 259,000 g·mol^−1^ and polydispersity index (PDI) of 1.94 was purchased from NatureWorks (Plymouth, MN, USA). Polyethylene glycol (PEG) with Mw = 100,000 g·mol^−1^ was supplied by Serva (Heidelberg, Germany). Quercetin (QUE, purity ≥95%) came from Sigma-Aldrich (St. Louis, MO, USA). Rosmarinic acid (RA) and the necessary buffer salts were provided by Merck (Billerica, MA, USA). All organic solvents—dichloromethane (DCM), absolute ethanol (EtOH), and dimethyl sulfoxide (DMSO)—were of analytical grade and used without further purification. The stable radical 2,2-diphenyl-1-picrylhydrazyl (DPPH) was used to assess antioxidant activity.

For cell culture experiments, Dulbecco’s Modified Eagle Medium (DMEM) and fetal bovine serum (FBS) were purchased from Gibco-Invitrogen (Leicestershire, UK). Penicillin–streptomycin solution was obtained from Lonza (Verviers, Belgium). Dimethyl sulfoxide (DMSO), Trypsin-EDTA solution (2.5 g/L trypsin, 0.2 g/L EDTA), and phosphate-buffered saline (PBS) were supplied from AppliChem (Darmstadt, Germany). The MTT reagent (3-[4,5-dimethylthiazol-2-yl]-2,3-diphenyl tetrazolium bromide) was sourced from Sigma-Aldrich Chemie GmbH (Darmstadt, Germany). Fluorescent stains acridine orange and ethidium bromide were purchased from Merck (Darmstadt, Germany). All sterile plastic consumables used were provided by Orange Scientific (Braine-l’Alleud, Belgium).

### 2.2. Electrospun Scaffold Fabrication

Polymer solutions for electrospinning were formulated from PLA, PLA/PEG blends, and their respective variants loaded with the polyphenols QUE and RA. A solvent system of dichloromethane and ethanol (80:20 *v*/*v*) was used to dissolve PLA and PLA/PEG (80:20 *w*/*w*) under constant stirring, yielding homogeneous 10 wt.% polymer solutions. Optimization studies carried out in advance established the PLA/PEG ratio selected for this study [33,39].

Polyphenols were added to the polymer solutions at defined concentrations. For single-component systems (PLA/RA, PLA/PEG/RA, PLA/QUE, and PLA/PEG/QUE), 10 wt.% of the respective polyphenol relative to the total polymer mass was used. In the case of dual-loaded systems (PLA/QUE/RA and PLA/PEG/QUE/RA), each compound was added at 5 wt.%.

The electrospinning setup consisted of a high-voltage power supply connected to a 20 G stainless steel needle attached to a 12 mL syringe. Process conditions included a 25 kV potential difference and a 12 cm gap to the rotating collector (1000 rpm). An NE-300 Just Infusion™ syringe pump (New Era Pump Systems Inc., Farmingdale, NY, USA) maintained the solution flow at 3 mL·h^−1^. Post-processing, vacuum treatment at room conditions ensured the thorough removal of solvent traces.

Following fabrication, the electrospun mats were dried under vacuum at room temperature to ensure complete removal of residual solvents.

### 2.3. In-Depth Evaluation of Electrospun Fiber Morphology and Properties

Scanning electron microscopy (SEM) served as the primary tool for evaluating the surface architecture of the electrospun scaffolds. Prior to analysis, a 60 s gold sputter-coating was applied via a Jeol JFC-1200 coater (Tokyo, Japan) to improve surface conductivity. The samples were then examined with a Jeol JSM-5510 microscope (JEOL Co., Ltd., Tokyo, Japan). Fiber dimensions were derived from the SEM micrographs using ImageJ (version 1.54g); specifically, a minimum of 30 random fibers were measured to calculate the mean diameter and standard deviation.

The solid-state structure of the composites was analyzed via X-ray diffraction (XRD). Diffractograms were obtained using a D8 Advance diffractometer (Bruker, Billerica, MA, USA) equipped with CuKα radiation and a position-sensitive detector. The scanning parameters included a 2θ range of 10–60°, a step size of 0.02°, and a dwell time of 1 s per step.

The mechanical performance of the electrospun non-wovens was analyzed via a universal testing system (Instron 3344, Instron, Norwood, MA, USA) equipped with a 50 N load cell and operated via Bluehill Universal software (version 3.11). Measurements were performed at ambient temperature (21 °C) with a crosshead speed of 10 mm·min^−1^. Rectangular specimens with dimensions of 20 mm × 60 mm and an approximate thickness of 350 µm were tested. For each sample, a minimum of 10 specimens was analyzed. The mechanical parameters, including elongation at break (εB, %), tensile strength (σ, MPa), and Young’s modulus (E, MPa), were determined from the linear region of the corresponding stress–strain curves, and average values were reported.

Quercetin and rosmarinic acid content in the fibrous materials was determined by dissolving samples (1 cm^2^) in 10 mL of dichloromethane/ethanol (80/20 *v*/*v*). Then the absorbance at 373 nm and 321 nm was measured using a DU 800 spectrophotometer UV (Beckman Coulter, Brea, CA, USA). The experiments were repeated three times.

### 2.4. Evaluation of Antioxidant Properties of Fibrous Systems

The DPPH method was utilized to assess the radical-neutralizing potential of quercetin (QUE) and the electrospun fibrous mats. The hydrogen-donating capacity of QUE was assessed by tracking the color shift in a DPPH ethanol solution from violet to yellow, a visual indicator of antioxidant activity. To evaluate the antioxidant activity, ethanol solutions of QUE and RA (5 × 10^−3^ M) and fibrous samples (PLA, PLA/PEG, PLA/QUE, PLA/RA, PLA/PEG/QUE, PLA/PEG/RA, PLA/QUE/RA, and PLA/PEG/QUE/RA; 0.5 mg each) were incubated for 30 min at room temperature in the dark with 3 mL of DPPH solution (1 × 10^−4^ M). After incubation, the remaining DPPH radicals were quantified by measuring the absorbance at 517 nm using a DU 800 UV–VIS spectrophotometer (Beckman Coulter, Brea, CA, USA). The antioxidant activity (AA%) was then calculated using the following equation:

$$\mathrm{Inhibition},\;\mathrm{AA},\%=\left[\frac{(A_{\mathrm{DPPH}}-A_{\mathrm{sample}})}{A_{\mathrm{DPPH}}}\right]\;\times \;100\;$$

where A_sample_–DPPH• is the absorbance of the DPPH solution after addition of the sample or flavonoid, and A_DPPH•_ is the absorbance of the control DPPH solution. All measurements were performed in triplicate.

### 2.5. Anticancer Potential

#### 2.5.1. Cell Lines and Culture Conditions

The human cervical carcinoma cell line HeLa (CCL-2) and the mouse embryo fibroblast cell line BALB/3T3 (CCL-163) were obtained from the American Type Culture Collection (ATCC, Rockville, MD, USA). Cell cultures were maintained in Dulbecco’s Modified Eagle Medium (DMEM) supplemented with 10% heat-inactivated fetal bovine serum (FBS), 2 mM L-glutamine, 50 U/mL penicillin, and 50 µg/mL streptomycin. Cells were cultivated in 25 cm^2^ tissue culture flasks at 37 °C in a humidified atmosphere containing 5% CO_2_, and were routinely passaged every 3–4 days to ensure exponential proliferation. When cultures reached 60–80% confluence, cells were detached using 0.25% trypsin-EDTA (pH 7.4), counted, and then resuspended in fresh growth medium at the required densities for subsequent assays.

#### 2.5.2. Cytotoxicity Assessment

The cytotoxic potential of the developed fibrous mats was evaluated using the MTT colorimetric assay. HeLa cervical cancer cells, along with normal fibroblasts BALB/3T3, were plated in 96-well cell culture plates at a density of 1 × 10^4^ cells per well. Following a 24 h incubation period at 37 °C and 5% CO_2_ to allow cell attachment and monolayer formation, the cells were exposed to the different fibrous samples for 24 and 72 h. Mats with 1.75 mg/0.28 cm^2^ were sterilized by UV irradiation for 15 min prior to cell culture experiments and then placed into wells containing 100 µL of culture medium. Cells cultured in medium alone, as well as those treated with quercetin (QUE, 100 µM) or rosmarinic acid (RA, 100 µM) solutions, served as controls. After treatment, the fibrous mats and medium were discarded, and 0.5 mg/mL MTT solution was added to each well, followed by incubation for 3 h at 37 °C to allow formation of formazan crystals. The MTT-containing medium was then removed, and a lysis solution consisting of DMSO/ethanol (1:1) was added to solubilize the resulting formazan crystals. Absorbance was recorded at 570 nm using a microplate reader (TECAN, Sunrise™, Grödig/Salzburg, Austria). Each test material was assayed in five replicates, and results were presented as mean ± standard deviation (SD) from three independent experiments. Cell viability was calculated using the following formula:

Cell viability (%) = (OD_experimental_/OD_control_) × 100

#### 2.5.3. Acridine Orange/Ethidium Bromide Fluorescent Staining for Cell Death Analysis

Double fluorescent staining with acridine orange and ethidium bromide (AO/EtBr) was used to assess cell viability and detect morphological changes associated with cell death, following 24 h of exposure to the tested fibrous mats. Acridine orange penetrates both live and dead cells and emits green fluorescence upon binding to DNA, indicating viable cells. Ethidium bromide selectively enters non-viable cells with compromised membrane integrity and stains the nucleus red, producing red fluorescence, marking dead or late apoptotic cells. For the assay, cancer and noncancerous cells were plated onto sterile glass coverslips placed in 24-well cell culture plates at a density of 2.0 × 10^5^ cells per well and cultured for 24 h in a CO_2_ incubator to allow cell adhesion and monolayer formation. Subsequently, the cells were treated with the tested samples. Mats with 1.75 mg/0.28 cm^2^, pre-sterilized by UV irradiation, were placed into wells containing 1 mL of culture medium. Untreated cells maintained in culture medium served as controls. After an additional 24 h incubation, the mats were removed, coverslips were collected, washed with phosphate-buffered saline (PBS), and stained with a fluorescent dye solution containing 5 µg/mL AO and 5 µg/mL EtBr. The fluorescently stained cells were immediately examined under a fluorescence microscope, Leica DM 5000B (Leica Microsystems, Wetzlar, Germany), to distinguish live (green), early apoptotic (green with nuclear condensation), and late apoptotic or necrotic cells (orange/red fluorescence).

#### 2.5.4. DAPI Fluorescent Staining for Nuclear Morphology Assessment

The morphology of the nucleus was evaluated employing the DNA-binding fluorescent dye 4′,6-diamidino-2-phenylindole dihydrochloride (DAPI). This dye can penetrate intact cell membranes, readily binds to DNA, and enables clear visualization of nuclear structures in both live and fixed cells.

Cancer and noncancerous cell lines were grown on sterile glass coverslips and then treated with the respective formulations as described above. After 24 h, the cells were fixed with methanol and subsequently stained with 1 µg/mL DAPI solution (in methanol) for 15 min at 37 °C in the dark. The coverslips with the stained cells were then mounted onto microscope slides using Mowiol^®^ mounting medium. The samples were examined under a fluorescence microscope (Leica DM 5000B, Wetzlar, Germany) to observe nuclear morphology and identify features such as chromatin condensation and nuclear fragmentation.

### 2.6. Statistical Analysis

All data are presented as mean values ± standard deviation (SD). Statistical analysis was performed using one-way analysis of variance (ANOVA) and consequent Bonferroni’s post hoc test for multiple comparisons. Data were statistically analyzed using GraphPad Prism version 5.0 (GraphPad Software Inc., San Diego, CA, USA). Differences were considered statistically significant at *p* < 0.05, and significance levels were indicated as follows: * *p* < 0.05, ** *p* < 0.01, and *** *p* < 0.001.

## 3. Results and Discussion

The proposed strategy in this work focuses on integrating biocompatible poly(lactic acid) (PLA) and water-soluble polyethylene glycol (PEG), both characterized by low toxicity, as effective carriers for quercetin (QUE) and rosmarinic acid (RA), both natural polyphenolic compounds. RA is known for its antioxidant, anticancer, and antimicrobial properties, positioning it as a strong candidate for biomedical use. Similarly, QUE exhibits strong antioxidant, anti-inflammatory, anticancer, and antimicrobial activities, further enhancing its potential in therapeutic applications. Encapsulation of these bioactive compounds within the selected polymer matrices is expected to improve their aqueous solubility and bioavailability, while also maintaining their stability and functional activity during storage.

Our previous studies reveal that electrospinning of 10 wt.% solutions of poly(lactic acid) (PLA) and PLA/poly(ethylene glycol) (PLA/PEG) resulted in the formation of continuous fibrous matrices with uniform morphology and no observable defects. The optimization of processing parameters played a key role in achieving consistent fiber formation, indicating stable jet behavior during electrospinning. The obtained fibrous structures exhibited high surface area and interconnected porosity, features that are advantageous for the incorporation and subsequent release of bioactive compounds [39].

The incorporation of quercetin and rosmarinic acid, both individually and in combination, did not compromise fiber integrity, suggesting good compatibility between the polyphenols and the polymer matrices. These compounds are well known for their antioxidant and therapeutic properties, and their successful integration into the fibrous systems supports the potential of these materials as delivery platforms. The combined loading approach was further considered in terms of possible synergistic interactions, which may enhance the overall biological performance of the system.

The present findings are consistent with previous reports on PLA-based electrospun carriers. Quercetin-loaded PLA fibers have been shown to improve compound stability and provide sustained release, leading to enhanced anticancer activity in various cell models [34,40]. Similarly, PLA systems containing rosmarinic-acid-rich plant extracts have demonstrated significant antioxidant activity along with controlled release behavior, confirming the suitability of these matrices for hydrophilic bioactives [33]. In addition, electrospun PLA materials incorporating plant-derived extracts have exhibited combined antimicrobial and antioxidant effects, reinforcing their multifunctional character [41,42].

### 3.1. Morphology of the Fibrous Mats

**Figure 1:**
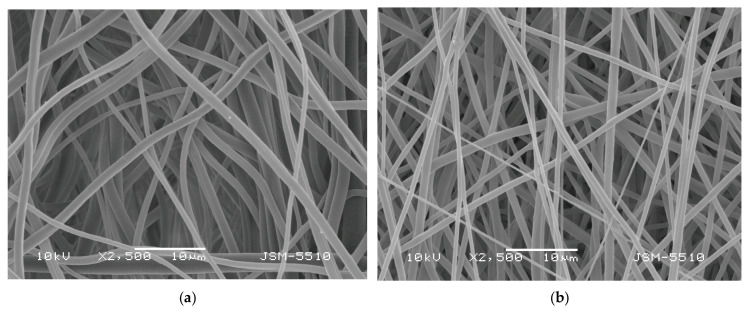

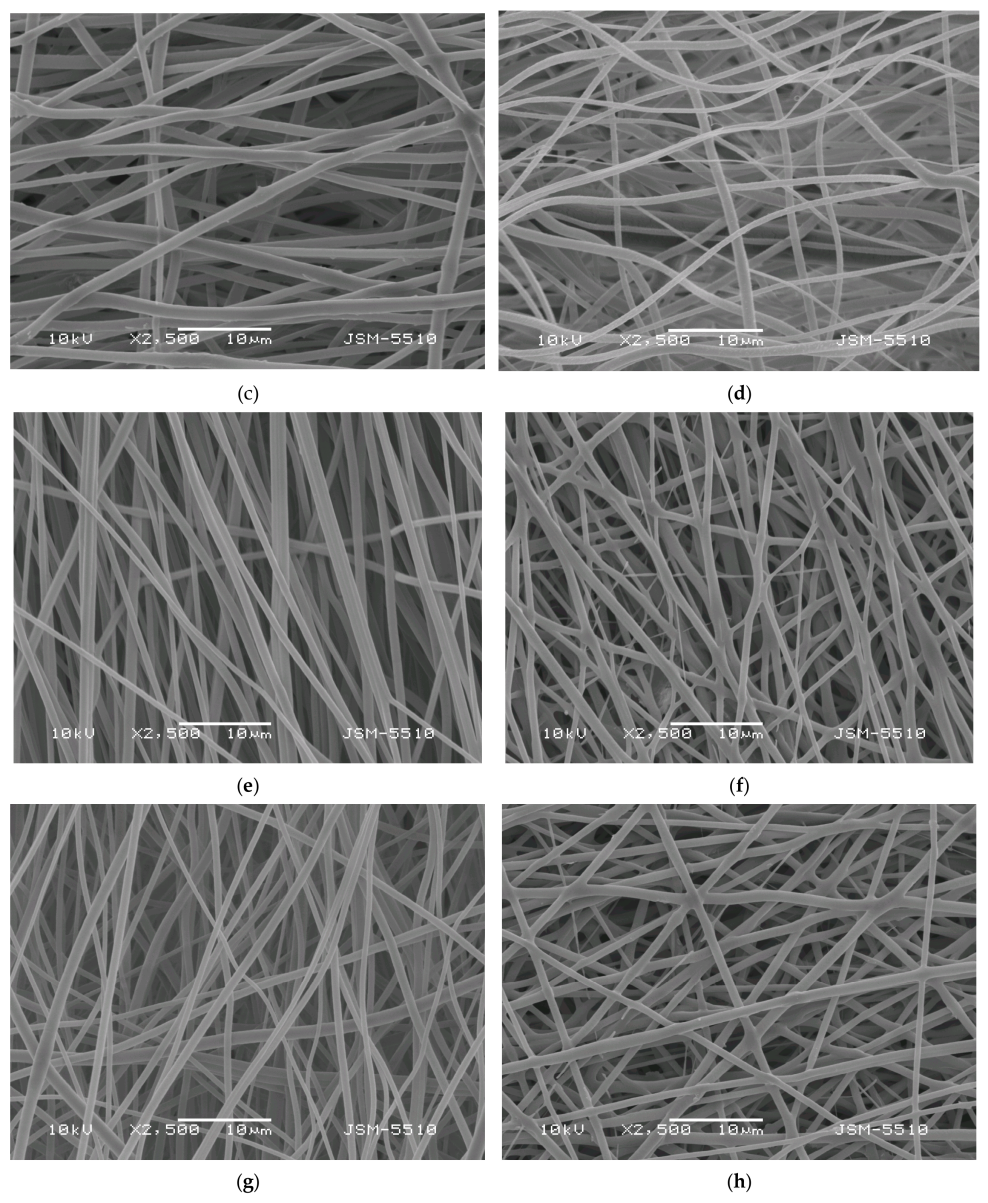
SEM images of electrospun materials: (**a**) PLA, (**b**) PLA/PEG, (**c**) PLA/QUE, (**d**) PLA/PEG/QUE, (**e**) PLA/RA, (**f**) PLA/PEG/RA, (**g**) PLA/QUE/RA, and (**h**) PLA/PEG/QUE/RA. Magnification ×2500.

Scanning electron microscopy (SEM) was used to observe the morphology of the obtained fibrous mats. SEM images are presented in Figure 1. PLA fibers have diameters of 768 ± 138 nm (Figure 1a). The introduction of polyethylene glycol (PEG) into the PLA matrix had a noticeable influence on the fibers’ diameters. The measured PLA/PEG fiber diameters were 671 ± 137 nm (Figure 1b). The detected reduction in the value is due to the lower molecular weight of the hydrophilic PEG. The mean fiber diameter of PLA/QUE and PLA/PEG/QUE fibers was insignificantly increased to 807 ± 159 nm and 713 ± 143 nm, respectively (Figure 1c,d). The fibers of PLA and PLA/PEG loaded with RA and QUE/RA have similar values to those loaded with QUE. The mean fiber diameters of PLA/RA (Figure 1e), PLA/PEG/RA (Figure 1f), PLA/QUE/RA (Figure 1g), and PLA/PEG/QUE/RA (Figure 1h) were 846 ± 149 nm, 720 ± 144 nm, 974 ± 148 nm, and 826 ± 152 nm, respectively. The QUE and RA loading into the fibers resulted in a slight enhancement of the PLA and PLA/PEG fiber diameters. This is probably due to the increase in viscosities after adding the flavonoids to the polymer solutions.

### 3.2. X-Ray Diffraction Characterization

**Figure 2:**
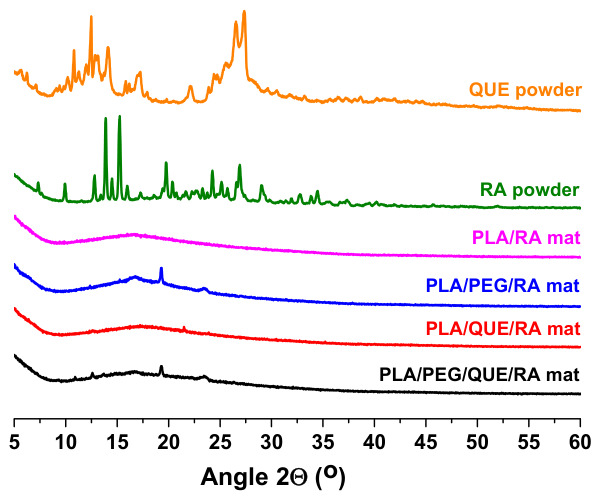
X-ray patterns of QUE (powder) and RA (powder), as well as of fibrous PLA/RA, PLA/PEG/RA, PLA/QUE/RA, and PLA/PEG/QUE/RA materials.

X-ray diffractometry was employed to examine the solid-state organization of the pure compounds and the electrospun composites. Figure 2 presents the XRD patterns of neat QUE and RA powders, as well as the electrospun PLA/RA, PLA/PEG/RA, PLA/QUE/RA, and PLA/PEG/QUE/RA mats. The diffractograms of all samples were recorded over a 2θ range of 5° to 60°. A well-ordered crystal lattice was evident in the diffractogram of neat quercetin (QUE) powder, which showed a number of sharp, intense reflections. The strongest signals were found at 2θ values of 13.56–14.3°, 15.7–18.0°, 23.4°, and 27.3°, indicating that the flavonoid is highly crystalline. Similarly, rosmarinic acid (RA) powder generated sharp diffraction peaks at 13.7°, 15.2°, 19.8°, and 27.0°, which are in full agreement with published data and confirm its crystalline state [43,44]. When incorporated into the PLA-based non-woven mats via electrospinning, both polyphenols—QUE and RA—lost their characteristic crystalline reflections and were no longer discernible in the respective diffractograms. This observation indicates that, under the electrospinning conditions, both bioactive compounds undergo a transition from an ordered crystalline state to a disordered, amorphous form within the polymer fibers. The rapid evaporation of the solvent during the electrospinning process is believed to be the primary reason for this transformation; the short time available does not allow the polyphenol molecules to arrange into a crystal lattice. Consequently, the originally crystalline QUE and RA remain “frozen” in an amorphous dispersion inside the fibrous matrix—a highly desirable feature for biomedical applications where fast dissolution and release are needed. In the ternary (PLA/QUE/RA and PLA/PEG/RA) and quaternary (PLA/PEG/QUE/RA) fibrous systems, the characteristic diffraction peaks attributed to QUE and RA are no longer distinguishable in the XRD patterns. This suggests a transition towards an amorphous state for both compounds following their co-incorporation into the electrospun fibers. Moreover, the XRD pattern of the PLA and PLA/PEG fibrous mats obtained by electrospinning is shown in Supplementary Materials (Figure S1), revealing that the electrospun polymers were in an amorphous state as well.

### 3.3. Physico-Mechanical Properties

**Figure 3:**
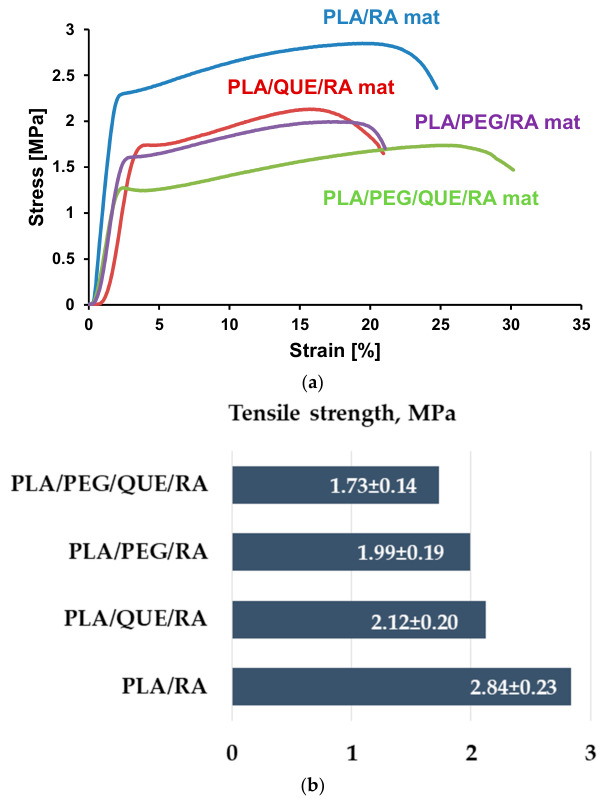
Mechanical characteristics of PLA/RA, PLA/QUE/RA, PLA/PEG/RA, and PLA/PEG/QUE/RA mats with a sample size of 20 mm × 60 mm × 350 µm. (**a**) Stress–strain curves and (**b**) tensile strength of the electrospun mats.

The mechanical performance of electrospun materials plays a critical role in dictating their functional viability and structural stability in biomedical applications such as advanced wound dressing, tissue engineering, and localized cancer therapy. The mechanical characteristics of electrospun polymer materials are highly dependent on processing conditions, individual fiber diameter, orientation, inter-fiber bonding, as well as the integration of plasticizing agents or low-molecular-weight bioactive molecules. In the present study, the mechanical behavior of the PLA/RA, PLA/QUE/RA, PLA/PEG/RA, and PLA/PEG/QUE/RA mats was evaluated via uniaxial tensile testing. The results were presented in Figure 3. Moreover, the mechanical properties of PLA, PLA/PEG, PLA/QUE, and PLA/PEG/QUE were determined in our previous study [35]. Pure electrospun PLA mats exhibited the highest tensile strength (3.36 ± 0.32 MPa) and Young’s modulus of 206 ± 14.68 MPa. The RA-loading into the PLA mat resulted in a slight decrease in the mat’s mechanical characteristics (tensile strength—2.84 ± 0.21 MPa). The incorporation of 20 wt.% PEG into the PLA matrix altered the mechanical behavior of the fibrous PLA mats as well. For the PLA/PEG mats, a decrease in tensile strength to 2.47 ± 0.19 MPa and Young’s modulus to 139.8 ± 10.23 MPa was detected. This modification is attributed to the low molecular weight of PEG. The incorporation of low-molecular-weight polyphenolic bioactives—quercetin and rosmarinic acid—exerted a distinct impact on the mechanical profile of both PLA and PLA/PEG mats. Loading the polyphenols into the polymer fibrous mats led to an additional slight reduction in tensile strength. The lowest mechanical characteristics showed the mat composed of PLA with the addition of a low-molecular-weight PEG and loaded with a combination of the two polyphenols (tensile strength—1.73 ± 0.14 MPa). Nevertheless, this mechanical profile remains well-suited for its intended application in localized, non-load-bearing soft-tissue oncology patches or postoperative antitumor coatings. In biomedical applications involving soft tissues—such as the human skin, bladder, or internal organs—the required physiological tensile strength typically ranges from 1 to 5 MPa. Therefore, a tensile strength of 1.73 MPa ensures that the nanofibrous membrane possesses adequate structural integrity to be surgically handled and sutured or applied onto soft internal surfaces without suffering premature tearing, while remaining flexible enough to conform to anatomical geometries. The obtained results ensure that the functionalized hybrid nanofibers can easily withstand physical manipulation during application without premature fracturing or structural failure.

### 3.4. Release Behavior and Encapsulation Efficiency

The release behavior of QUE and RA from electrospun PLA/QUE, PLA/RA, PLA/PEG/QUE, PLA/PEG/RA, PLA/QUE/RA, and PLA/PEG/QUE/RA mats was studied in detail at 37 °C in both acetate buffer and phosphate-buffered saline and is presented in detail in our previous study [33]. The release of QUE from PLA and PLA/PEG fibers was 60.5% ± 1.12% and 87.3% ± 1.18% (pH 5.5) and 55.3% ± 1.38% and 80.8% ± 1.45% (pH 7.4), respectively. Additionally, the release of RA from the same fibrous mats was 69.5% ± 0.98% and 90.2% ± 1.01% (pH 5.5) and 51.1% ± 1.39% and 88.4% ± 1.22% (pH 7.4), respectively. The results reveal that the highest release profile of QUE was observed from the PLA/PEG/QUE/RA mat: 96.8% ± 1.56% at pH 5.5 and 95.0% ± 1.17% at pH 7.4. Moreover, the highest release of RA was detected from the same mat, and it is 96.2% ± 1.37% and 86.8% ± 1.33% in a buffer solution with pH 5.5 and pH 7.4, respectively. This is due to the fact that PEG-induced hydrophilicity to the fibrous mats and the polyphenol loaded into the fibers could diffuse easily. The results for the release of QUE and RA from PLA/QUE, PLA/PEG/QUE, PLA/QUE/RA, and PLA/PEG/QUE/RA fibers were cross-checked by determining the encapsulation efficiency of QUE and RA into the polymer fibers. For this purpose, the fibrous mats were dissolved in dichloromethane/ethanol, and the absorbance of the obtained solution at 373 nm and 321 nm was recorded. It was found that the total amount of QUE and RA in the PLA/PEG fibrous mats was 96.8% and 96.2%, respectively. These results indicate that QUE and RA encapsulation efficiency was close to 100%.

### 3.5. Assessment of Antioxidant Capacity

The antioxidant activity of the prepared electrospun mats was primarily evaluated using the 2,2-diphenyl-1-picrylhydrazyl (DPPH) free radical scavenging assay. This method is one of the most widely used, simple, and rapid assays for determining the capacity of a compound or extract to act as a free radical scavenger or hydrogen donor. The assay relies on the principle that the stable DPPH radical, which is characterized by its deep violet/purple color in solution and strong absorbance at approximately 517 nm, is reduced upon encountering an antioxidant. The antioxidant donates a hydrogen atom or electron, converting the radical to its non-radical, pale yellow form (DPPH-H), with the degree of color loss being proportional to the radical-scavenging activity of the sample and quantified spectrophotometrically [45].

**Figure 4:**
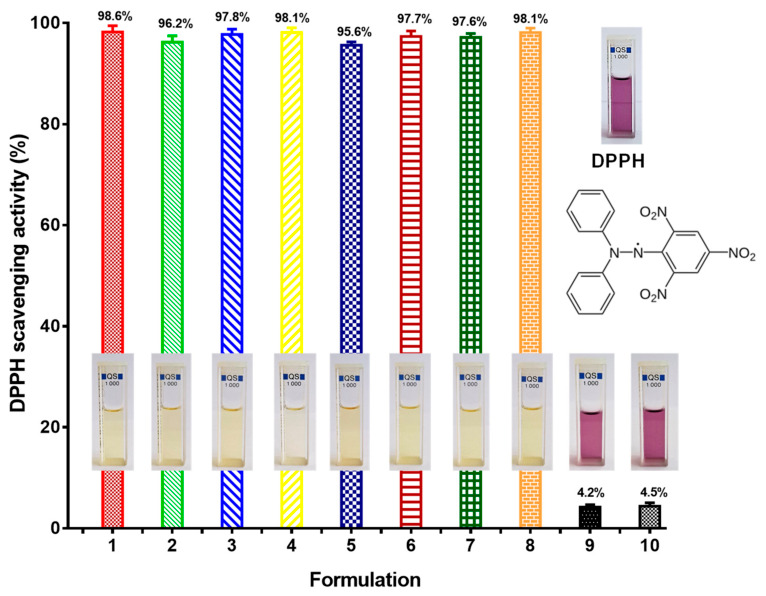
Antioxidant activity of 1—ethanol solution of RA, 2—PLA mat containing 10 wt.% RA, 3—PLA/PEG mat containing 10 wt.% RA, 4—ethanol solution of QUE, 5—PLA mat containing 10 wt.% QUE, 6—PLA/PEG mat containing 10 wt.% QUE, 7—PLA mat containing 5 wt.% RA and 5 wt.% QUE, 8—PLA/PEG mat containing 5 wt.% RA and 5 wt.% QUE, 9—PLA mat, and 10—PLA/PEG mat. Results represent the mean ± SD of *n* = 3 independent experiments performed in triplicate.

The choice of rosmarinic acid (RA) and quercetin (QUE) as active agents was motivated by their robust and well-documented antioxidant profiles. Rosmarinic acid (RA), a naturally occurring phenolic acid, is well known for its strong antioxidant, anti-inflammatory, and neuroprotective effects. Its efficacy as a direct free radical scavenger is primarily attributed to its molecular structure, which includes multiple hydroxyl groups that significantly enhance its capacity to donate electrons and neutralize reactive oxygen species (ROS) [16]. Similarly, quercetin (QUE), a major flavonoid, is globally recognized as a formidable antioxidant and is frequently employed as a positive control in antioxidant studies due to its high potency [46]. Studies comparing these compounds in non-cellular systems, such as the DPPH assay, have indicated that the radical-scavenging activity of RA is comparable to that of QUE [47]. The overall free-radical scavenging capabilities of both polyphenols stem from their highly conjugated systems and distinct hydroxylation patterns, which allow them to effectively intercept and stabilize free radicals. As anticipated, the reference standards confirmed the inherent high activity of the pure compounds, with ethanol solutions of RA and QUE exhibiting radical scavenging efficiencies of 98.6% and 98.1%, respectively (Figure 4(1) and Figure 4(4)). Importantly, the process of electrospinning successfully preserved the bioactivity of these molecules upon their incorporation into the polymer matrix. The PLA mats containing a single active compound at a 10 wt.% loading demonstrated strong activities: the PLA/RA mat achieved 96.2% activity, and the PLA/QUE mat achieved 95.6% DPPH scavenging.

A notable finding was the positive influence of PEG on the mats’ performance. The inclusion of PEG in the polymer blend furthermore improved the antioxidant activity of the materials, which is likely due to an enhancement in the hydrophilicity of the fibrous structure, facilitating a more effective release and accessibility of the active compounds within the assay environment. Specifically, the PEG-containing mats, PLA/PEG/RA and PLA/PEG/QUE, achieved antioxidant efficiency of 97.8% and 97.7%, respectively. Furthermore, the formulations that combined both antioxidants (each at 5 wt.%) maintained exceptional activity. The PLA/RA/QUE mat reached a high scavenging efficiency of 97.6% (Figure 4(7)), while the four-component composite, PLA/PEG/RA/QUE, exhibited the highest efficiency observed among all tested fibrous systems at 98.1% (Figure 4(8)). It must be noted, however, that at these high percentages, the antioxidant activity is near saturation, and the minor numerical variations between the active formulations do not represent a statistically or practically significant difference. Therefore, the DPPH assay demonstrates excellent overall radical scavenging capacity but cannot be used to infer an enhanced or synergistic chemical interaction between the two polyphenols due to this plateau effect. This result suggests a beneficial combined effect between RA and QUE when acting in concert, which may be attributed to their complementary pathways of radical neutralization rather than formal mathematical synergy. Visual confirmation of the strong antioxidant effects was observed in the DPPH assay, where all active mats induced a clear color change from the initial purple hue of the control solution to a pale yellow (Figure 4). In contrast, the control samples—PLA and PLA/PEG mats, which contained no active compounds—registered minimal scavenging activity of 4.2% and 4.5% (Figure 4(9) and Figure 4(10)), respectively. These negligible values confirmed that the robust radical-scavenging effect is exclusively attributable to the presence of RA and/or QUE within the composite materials. Statistical analysis further validated these results, showing that the improvements in antioxidant activity for the active composite mats were all highly significant (*p* < 0.001) compared to the control groups. These findings highlight the strong potential of PLA-based electrospun mats loaded with natural antioxidants for biomedical applications where oxidative stress management is essential, such as wound healing, tissue regeneration, or active packaging systems.

### 3.6. In Vitro Assessment of Anticancer Potential

Electrospun nanofibers possess several advantageous characteristics, including high drug-loading capacity, large specific surface area, high porosity, efficient encapsulation, facile surface functionalization, compatibility with multiple therapeutic modalities, cost-effectiveness, and overall versatility. Owing to these unique structure-related properties, they offer significant potential for applications in cancer diagnosis and treatment, including highly sensitive point-of-care biosensing platforms, selective cancer cell capture technologies, and advanced functionalized drug delivery systems for targeted anticancer therapy [48]. Given their promising role as drug delivery platforms, the biological performance of the developed electrospun nanofibers was evaluated through a series of in vitro assays. Particular attention was paid to their anticancer activity and biocompatibility, as these parameters are critical for their potential therapeutic application.

#### 3.6.1. Assessment of Cytotoxicity

**Figure 5:**
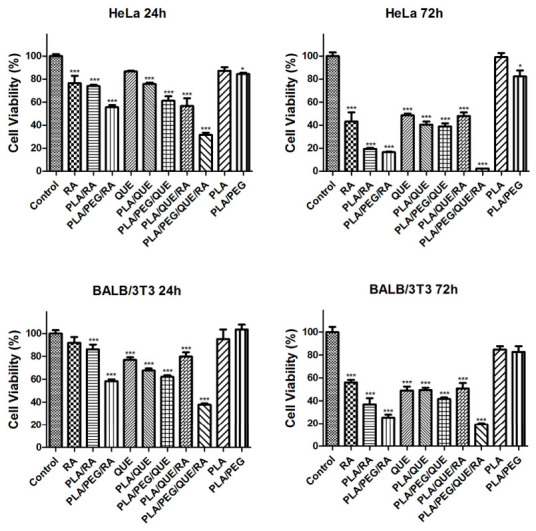
Effect of the different formulations on cell viability of HeLa and BALB/3T3 cells after 24 h and 72 h of exposure. Cell viability was evaluated by the MTT assay and expressed as a percentage of the untreated control. Data are expressed as mean ± SD (n = 5). Statistical significance compared to the control is denoted as * *p* < 0.05, and *** *p* < 0.001.

The cytotoxicity of each formulation was assessed in HeLa and BALB/3T3 cell lines after 24 and 72 h of exposure (Figure 5).

HeLa cells exhibited a distinct time-dependent reduction in viability for most treatments. After 24 h, RA and PLA/RA mats induced a moderate decrease in viability (76.5% and 74.0%, respectively), whereas PLA/PEG/RA resulted in a more pronounced reduction (55.6%; *** *p* < 0.001, compared to RA and PLA/RA). QUE and QUE-loaded mats (PLA/QUE and PLA/PEG/QUE) reduced viability to 86.7%, 75.9%, and 61.3%, respectively. The treatment with fibrous mats loaded with both bioactive compounds (PLA/RA/QUE and PLA/PEG/RA/QUE) induced the greatest reduction in viability (56.7% and 31.5%, respectively). In contrast, cells treated with PLA and PLA/PEG mats retained relatively high cell viability (~80–90%), indicating low intrinsic toxicity of the carrier systems.

After 72 h, the cytotoxic effects were significantly enhanced. RA reduced HeLa cell viability to 43.2%, while PLA/RA and PLA/PEG/RA were more active and significantly decreased (*** *p* < 0.001, compared to RA) viability to 19.5% and 16.6%, respectively. QUE-based formulations (PLA/QUE and PLA/PEG/QUE) demonstrated moderate to strong cytotoxicity (40.5% and 40.0% cell viability). The combined systems (PLA/RA/QUE and PLA/PEG/RA/QUE) showed the most pronounced cytotoxicity, with PLA/PEG/RA/QUE reducing cell viability to below 10%. As observed at 24 h, PLA and PLA/PEG alone remained largely non-toxic, maintaining high viability levels (~80–100%).

In BALB/3T3 cells, the reduction in viability was less pronounced compared to HeLa cells at both time points. After 24 h, most treatments, including RA, PLA/RA, and QUE-based systems, maintained cell viability within the range of 60–80%, while PLA/PEG/RA/QUE exhibited a stronger effect (38.0%; *** *p* < 0.001, compared to PLA/PEG/RA and PLA/PEG/QUE). At 72 h, RA decreased the cell viability to 56.1%, while its incorporation into fibrous mats in PLA/RA and PLA/PEG/RA further reduced viability to 25–35%. In contrast, the differences in cytotoxicity of free QUE and QUE-containing formulations were less pronounced and showed moderate effects (~40–50% cell viability). The combined formulation PLA/PEG/RA/QUE again demonstrated the highest cytotoxicity 19.2% viability), although normal cells remained comparatively more resistant than cancer cells. Importantly, PLA and PLA/PEG maintain high cell viability (~80–95%), confirming their good biocompatibility.

The results of cytotoxicity assessment indicate that the tested formulations exert pronounced, time-dependent effects, with combined formulations, particularly PLA/PEG/RA/QUE, exhibiting the strongest antiproliferative activity. The higher sensitivity of HeLa cells compared to BALB/3T3 cells suggests a degree of selectivity toward cancer cells, while the minimal cytotoxicity and high biocompatibility of PLA and PLA/PEG mats alone support their suitability as effective drug delivery systems.

#### 3.6.2. Fluorescence Microscopy Analyses

**Figure 6:**
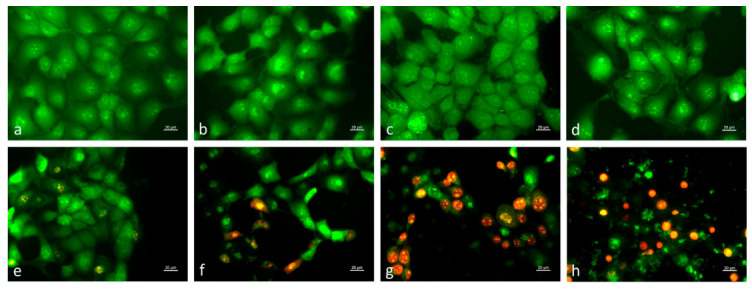
Fluorescence microscopy images of acridine orange/ethidium bromide stained HeLa cells treated for 24 h with different formulations: (**a**)—control; (**b**)—PLA/PEG mat; (**c**)—RA; (**d**)—QUE; (**e**)—PLA/RA mat; (**f**)—PLA/PEG/RA mat; (**g**)—PLA/QUE/RA mat; and (**h**)—PLA/PEG/QUE/RA mat. Scale bar: 20 µm.

To further analyze the effects of the electrospun mats on the viability and morphology of cancer cells, fluorescent microscopy analyses were performed after AO/EtBr staining (Figure 6). In this staining method, viable cells exhibit homogenous green fluorescence, whereas early apoptotic cells show intense green staining and chromatin condensation. Late apoptotic and necrotic cells show orange to red fluorescence due to increased ethidium bromide uptake.

Control cells exhibited uniform green fluorescence with intact morphology and well-defined nuclei, indicative of viable cells with preserved membrane integrity (Figure 6a). A similar pattern was observed in the PLA/PEG-treated cells, confirming the negligible cytotoxicity and good biocompatibility of the polymeric carrier (Figure 6b). Treatment with RA and QUE induced moderate apoptotic changes. Although most cells remained green-stained, slight nuclear condensation and a reduction in cell density were evident, suggesting the onset of early apoptosis (Figure 6c,d). The PLA/RA mats increased the number of cells exhibiting orange/yellow fluorescence, indicating a higher level of apoptosis compared to free RA (Figure 6e). A more pronounced effect was observed for the PLA/PEG/RA formulations, where a substantial number of cells displayed orange and red fluorescence, along with cell shrinkage and membrane blebbing—hallmarks of late apoptosis and necrosis (Figure 6f).

The most significant cytotoxic effects were observed for the combined RA- and QUE-containing formulations. PLA/QUE/RA treatment resulted in extensive apoptotic changes, such as chromatin condensation, nuclear fragmentation, and increased red/orange fluorescence (Figure 6g). These effects were even more pronounced in the PLA/PEG/QUE/RA, where the majority of cells exhibited intense red fluorescence, severe morphological disruption, and loss of normal cellular architecture, indicating advanced apoptosis and necrosis (Figure 6h).

**Figure 7:**
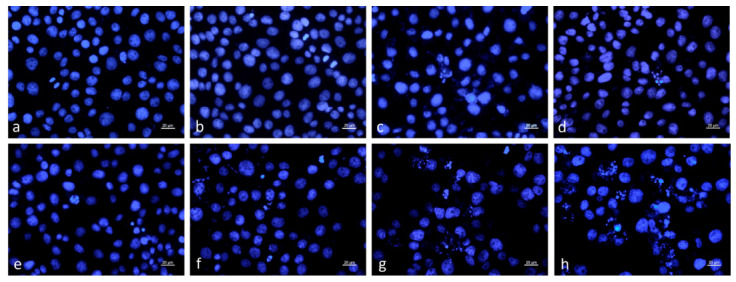
Fluorescence microscopy images of HeLa cells stained with DAPI after 24 h treatment with different formulations: (**a**)—control; (**b**)—PLA/PEG mat; (**c**)—RA; (**d**)—QUE; (**e**)—PLA/RA mat; (**f**)—PLA/PEG/RA mat; (**g**)—PLA/QUE/RA mat; and (**h**)—PLA/PEG/QUE/RA mat. Scale bar: 20 µm.

To additionally assess nuclear morphological changes induced by the tested fibrous mats in HeLa cancer cells, DAPI staining was performed (Figure 7).

The staining with DAPI showed distinct treatment-dependent alterations in nuclear morphology in HeLa cells, indicative of apoptosis and cell damage. Control and PLA/PEG-treated cells showed round, homogenously stained nuclei with intact chromatin, which is characteristic of viable cells, thereby confirming the low cytotoxicity and good biocompatibility of the carrier system (Figure 7a,b). Treatment with free RA and QUE resulted in slight chromatin condensation and occasional irregular nuclear morphology, suggesting the initiation of early apoptotic events (Figure 7c,d). More evident nuclear alterations, including chromatin condensation and the presence of smaller, brightly stained nuclei, were evident following exposure to PLA/RA (Figure 7e). These changes were even more pronounced in PLA/PEG/RA-treated cells, in which condensed and fragmented nuclei clearly indicated progression toward late apoptosis (Figure 7f). The most significant nuclear damage was observed in cells incubated in the presence of PLA/QUE/RA and PLA/PEG/QUE/RA mats (Figure 7g,h). These formulations induced extensive nuclear alterations, including highly condensed and fragmented nuclei, as well as the formation of apoptotic bodies—hallmarks of advanced apoptosis. Additionally, a marked reduction in cell density and disruption of nuclear integrity were evident. The DAPI staining demonstrated a progressive increase in apoptotic nuclear changes from single-agent treatments to combined formulations. The most pronounced effect was observed for PLA/PEG/QUE/RA, supporting its enhanced pro-apoptotic and antiproliferative activity in HeLa cells.

**Figure 8:**
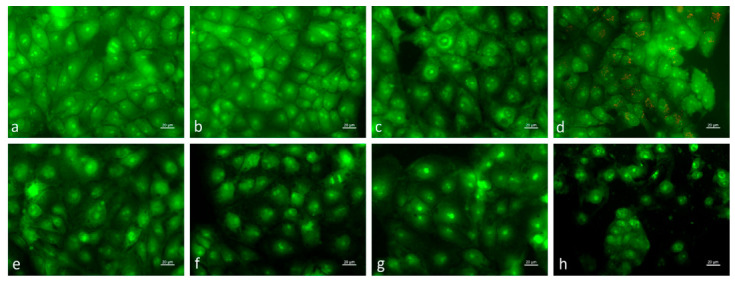
Representative fluorescence images of acridine orange/ethidium bromide stained BALB/3T3 cells after 24 h treatment with different formulations: (**a**)—control; (**b**)—PLA/PEG mat; (**c**)—RA; (**d**)—QUE; (**e**)—PLA/RA mat; (**f**)—PLA/PEG/RA mat; (**g**)—PLA/QUE/RA mat; (**h**)—PLA/PEG/QUE/RA mat. Scale bar: 20 µm.

**Figure 9:**
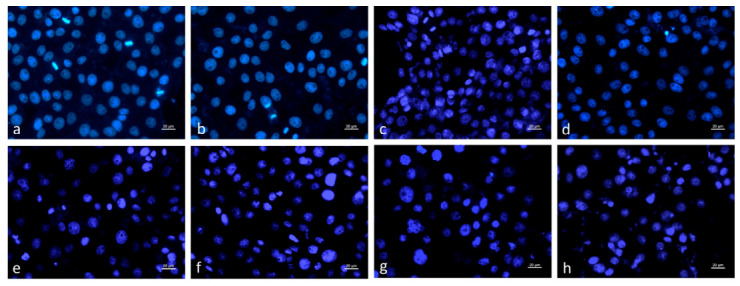
Fluorescence microscopy images of BALB/3T3 cells stained with DAPI after 24 h exposure to different formulations: (**a**)—control; (**b**)—PLA/PEG mat; (**c**)—RA; (**d**)—QUE; (**e**)—PLA/RA mat; (**f**)—PLA/PEG/RA mat; (**g**)—PLA/QUE/RA mat; and (**h**)—PLA/PEG/QUE/RA mat. Scale bar: 20 µm.

In addition to cancer cells, the morphological and nuclear changes were also evaluated in noncancerous cells to assess the selectivity and biocompatibility of the tested fibrous mats. The comparative analysis of AO/EtBr and DAPI stainings of BALB/3T3 cells reveals consistent and complementary insights into cell viability and nuclear integrity following treatment with the different fibrous formulations (Figure 8 and Figure 9).

The AO/EtBr-stained control and PLA/PEG-treated cells predominantly exhibited diffuse green fluorescent staining, indicative of viable cells with preserved membrane integrity (Figure 8a,b). Following treatment with RA and QUE, fluorescence microscopy analysis showed mainly green cells with occasional intense nuclear fluorescence, suggesting the occurrence of early apoptotic changes (Figure 8c,d). In the cell cultures treated with PLA/RA and PLA/PEG/RA formulations, a moderate increase in early apoptotic cells was found, although the majority of cells remained viable (Figure 8e,f). More pronounced effects were observed with the combined systems. Treatment with PLA/QUE/RA resulted in a higher proportion of early apoptotic cells showing intense green fluorescence (Figure 8g). The strongest effects were observed for PLA/PEG/QUE/RA mats, where AO/EtBr staining showed a marked reduction in cell density, cell shrinkage, and chromatin condensation, indicating early apoptotic changes (Figure 8h). However, in contrast to HeLa cells, BALB/3T3 cells retained their membrane integrity and showed green staining, suggesting greater resistance of normal cells. This observation was supported by the DAPI staining (Figure 9).

The untreated and PLA/PEG-treated cells appear round, homogenously stained, and structurally intact, confirming normal nuclear morphology and high cell viability (Figure 9a,b). In RA- and QUE-treated BALB/3T3 cells, DAPI staining revealed mild nuclear changes, including slight chromatin condensation and occasional irregularly shaped nuclei, indicative of early apoptotic events (Figure 9c,d). In cells exposed to PLA/RA and PLA/PEG/RA, increased nuclear condensation and occasional nuclear fragmentation were observed (Figure 9e,f). Cell cultures exposed to PLA/QUE/RA showed more frequent chromatin condensation and nuclear irregularities (Figure 9g). DAPI staining of PLA/PEG/QUE/RA-treated cells revealed the most severe nuclear changes, including condensation, fragmentation, and the presence of apoptotic bodies, but less pronounced than typically seen in cancer cells (Figure 9h).

Both staining methods consistently demonstrate that BALB/3T3 cells are less sensitive to all treatments than HeLa cancer cells. PLA, PLA/PEG mat show minimal cytotoxicity, while QUE- and RA-loaded formulations, and particularly PLA/PEG/QUE/RA, induce pronounced cellular and nuclear apoptotic changes. Results of fluorescent microscopy analysis provide strong evidence that apoptosis is the predominant mechanism of cell death induced by the tested electrospun mats. Together, these findings further support the selective cytotoxicity of the developed systems, showing reduced effects on normal cells alongside enhanced activity against cancer cells.

## 4. Conclusions

In this study, electrospinning was successfully employed to fabricate PLA and PLA/PEG fibrous materials loaded with quercetin (QUE) and rosmarinic acid (RA). All produced mats were free of structural defects. The fibers containing both polyphenols showed strong radical-scavenging ability. Notably, the four-component PLA/PEG/RA/QUE system reached the highest antioxidant efficiency among all tested formulations (98.1%), pointing to a potential synergistic effect between RA and QUE. Furthermore, the incorporation of QUE and RA conferred significant anticancer properties to the mats. The QUE- and RA-loaded PLA and PLA/PEG mats exerted strong antiproliferative activity against human cervical carcinoma cells HeLa and comparatively lower toxicity towards normal mouse BALB/3T3 fibroblasts. Overall, the electrospun PLA/PEG matrices displayed favorable mechanical performance, biocompatibility, and the capacity to host natural bioactive compounds. These features encourage further investigation of the developed fibrous systems in advanced biomedical contexts that demand antioxidant properties and tumor-targeting capabilities. The promising results obtained here suggest that these novel materials could be applied in wound healing and cancer therapy.

## Data Availability

The data are contained within this article.

## Supplementary Materials

The following supporting information can be downloaded at: https://www.mdpi.com/article/10.3390/biomimetics11070453/s1, Figure S1: X-ray patterns of: fibrous materials of PLA and PLA/PEG.
