# Supplementary figures and images for: Quercetin and Rosmarinic Acid Functionalized Hybrid Electrospun Nanofibers with Strong Antioxidant and Anticancer Activities
Source: Biomimetics (Basel). 2026 Jul 1;11(7):453. doi: 10.3390/biomimetics11070453 (PMC13406547; doi:10.3390/biomimetics11070453)

## Supplementary material

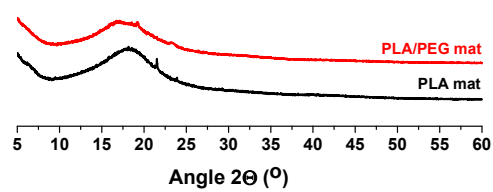

**Figure S1.** X-ray patterns of: fibrous materials of PLA and PLA/PEG.

Supplement: Supplementary file 1 [file biomimetics-11-00453-s001.zip › biomimetics-4377133-supplementary.pdf]
